# Supplementary figures and images for: Behavioral and genetic analysis of the effects of the psychedelic 2,5-dimethoxy-4-iodoamphetamine (DOI) in C. elegans
Source: PLoS One. 2025 Aug 26;20(8):e0329538. doi: 10.1371/journal.pone.0329538 (PMC12380321; doi:10.1371/journal.pone.0329538)

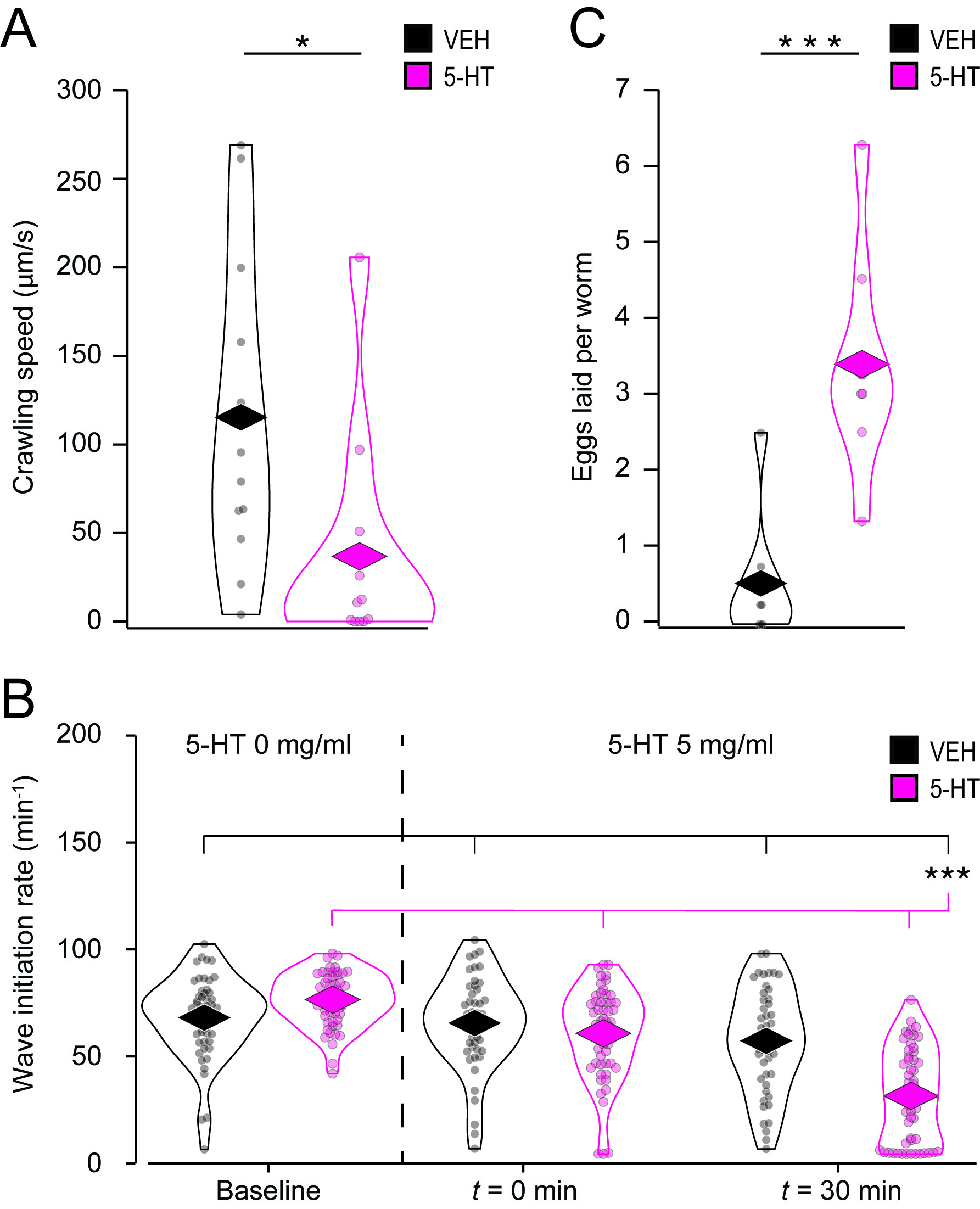

Supplement: S1 Fig — Animals were exposed to serotonin (5-HT) at 28 mM (5 mg/mL) or vehicle (VEH) for 30 min prior to testing. Diamonds, means. A. Crawling assay. Mean crawling speed is plotted versus condition. Each data point represents the mean of a cohort of 10−15 worms. n = 11 cohorts for 5-HT and 12 for VEH. *, two-sample t-test, p = 0.024. See S1 Table, row 5. B. Swimming assay. n = 52 individuals for VEH and n = 56 for 5-HT. ***, 2-way mixed ANOVA, interaction Time × 5-HT, p = 6.3 × 10^−11^. See S1 Table, row 6. C. Egg-laying assay. n = 8 cohorts for DOI and VEH (vehicle); ***, two-sample t-test, p = 5.16 x 10^−4^. See S1 Table, row 7. (TIF) [file pone.0329538.s001.tif]

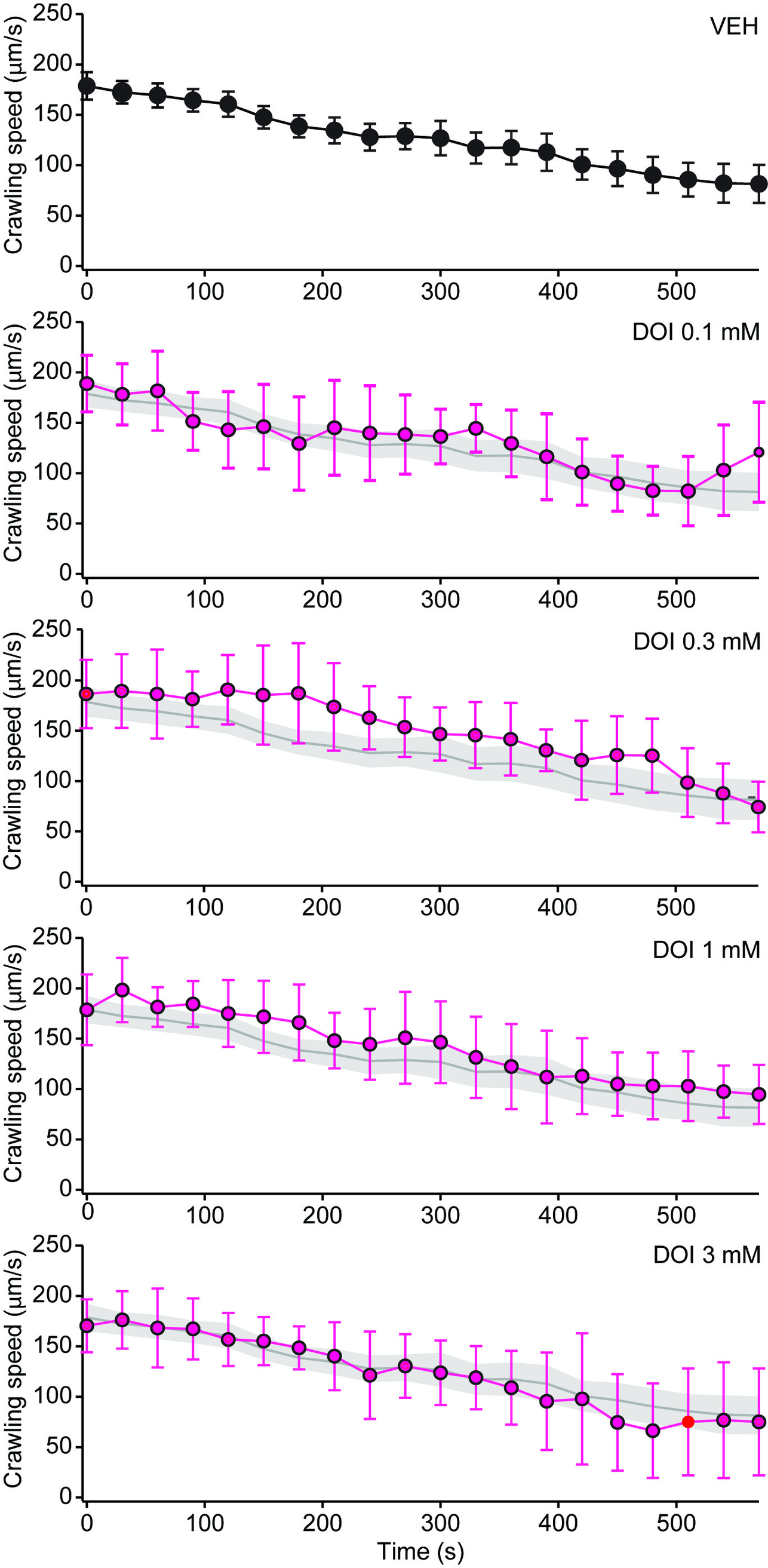

Supplement: S2 Fig — Each data point represents the mean speed in 30 sec. bins across cohorts of 10–15 worms. n = 8 cohorts at each dose of DOI and 31 for VEH (vehicle). DOI exposure time was 30 min. Error bars ± 95% CI. Shaded gray trace, VEH. Two-way ANOVA, dose versus time, main effect of time, p = 9.86 × 10^-34^, no effect of DOI dose, and no dose × time interaction. See S1 Table, row 8. (TIF) [file pone.0329538.s002.tif]

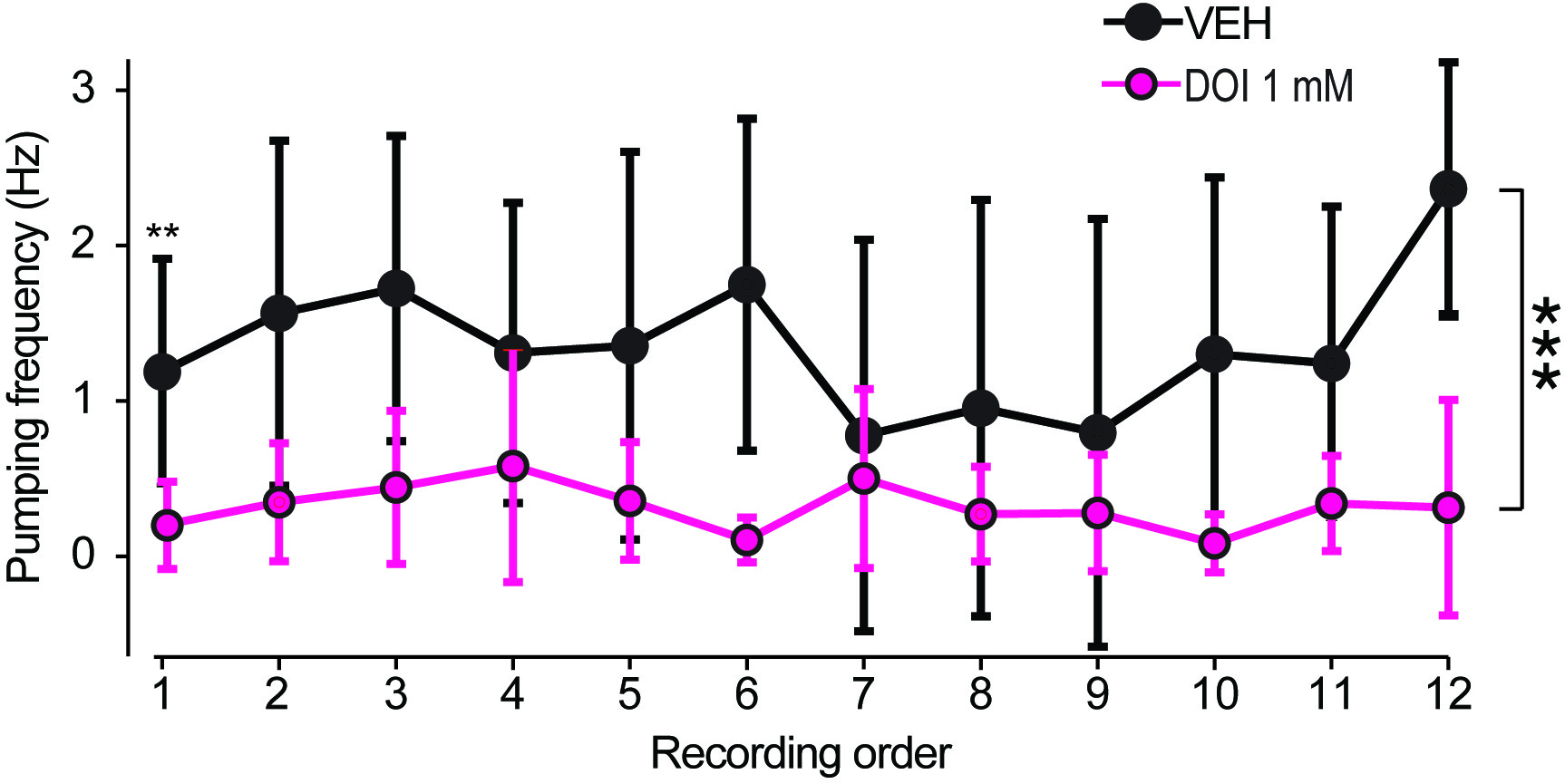

Supplement: S3 Fig — Same experiment as in Fig 4A. Recording order serves as a proxy for time in the recording device. Worms were exposed to DOI for 30−120 minutes, depending on the order in which they were recorded. Each point represents the mean pumping frequency for individual worms recorded at a particular rank in a series of 12 recordings. Error bars, ± CI; **, two-sample t-test, p = 0.016; •••, Two-way ANOVA order versus DOI, main effect of DOI, p = 6.16 × −10−10, no effect of order, no order × DOI interaction. See S1 Table, row 11,12. (TIF) [file pone.0329538.s003.tif]
